# The Co-Existence of Patent Omphalomesenteric Duct and Omphalocele in Patau’s Syndrome in Saudi Arabia: A Case Report

**DOI:** 10.7759/cureus.50793

**Published:** 2023-12-19

**Authors:** Badr Beyari, Yaser Alhassan, Aisha Gabra, Mohammed Awad, Ameen Alsaggaf

**Affiliations:** 1 Surgery, King Abdulaziz University Faculty of Medicine, Jeddah, SAU; 2 Orthopaedics, Royal College of Surgeons in Ireland, Dublin, IRL; 3 Pediatric Surgery, King Abdulaziz University Hospital, Jeddah, SAU; 4 Pediatric Surgery, Tanta University Faculty of Medicine, Tanta, EGY; 5 Pediatric Surgery, King Fahad Armed Forces Hospital, Jeddah, SAU

**Keywords:** palliative care, intestinal umbilical fistula, omphalomesenteric duct remnants, omphalocele, patau’s syndrome

## Abstract

The pathophysiology of Patau’s syndrome involves the triplication of chromosomes, leading to multiple comorbidities. An omphalocele is characterized by a protrusion of abdominal contents from the base of the umbilical cord through the peritoneum. An omphalomesenteric duct remnant occurs when there is a failure of duct closure that results in a diverticulum extending from the fetal midgut to the yolk sac. While congenital defects rarely occur simultaneously in patients with Patau’s syndrome, this case report describes a newborn with Patau syndrome who presented with both an omphalocele and an omphalomesenteric duct remnant. The newborn exhibited various congenital abnormalities such as coloboma, microphthalmia, broad nasal bridge, cleft lip, cleft palate, low-set ears, systolic murmur, omphalocele, intestinal umbilical fistula (omphalomesenteric continuous vitelointestinal duct remnant), polydactyly, rocker-bottom feet, left-sided clubbed foot, and ruptured myelomeningocele. Imaging revealed additional complications such as a large patent ductus arteriosus, hypoplastic distal arch, markedly dilated right atrium and left ventricle, and cerebellar hypoplasia. Chromosomal analysis confirmed the diagnosis of Patau’s syndrome. Given the untreatable medical condition, the patient was placed under “Do Not Resuscitate,” and palliative care was initiated. The simultaneous appearance of an omphalocele and an omphalomesenteric continuous vitelointestinal duct is rare, and surgical intervention is the standard of care if the patient is deemed suitable for surgery. However, in cases where surgery is not feasible, palliative care is initiated. Regardless of the outcome, genetic counseling is essential and should include a discussion on paternal autonomy, understanding the disorder, suggesting alternative management methods, and making crucial decisions concerning future family care and planning.

## Introduction

The pathophysiology of trisomy disorders is non-disjunction or Robersonian translocation, which causes a triplicate number of chromosomes rather than duplication [1]. There are different types of trisomy disorders, including Down’s syndrome (trisomy 21) and Edward’s syndrome (trisomy 18). Another example discovered by Patau et al. is Patau’s syndrome (trisomy 13) [2]. The prevalence of Patau’s syndrome is approximately one in 12,000 births in the United States, and it is typically considered fatal within the first year of life, with a 10% survival rate and a median survival rate of 7-10 days [1-3]. Patients with Patau’s syndrome present with severe intellectual disability, microphthalmia, cutis aplasia, polycystic kidney disease, holoprosencephaly, cleft lip and palate, low-set ears, polydactyly, congenital heart defects, rocker-bottom feet, and omphalocele [1,4]. An omphalocele is a congenital birth defect in which the abdominal contents protrude into the base of the umbilical cord and are covered only by a thin membrane [5]. Omphalomesenteric duct remnants are a spectrum of many conditions, ranging from cysts, fistulas, and bands to patent ducts, with the failure of duct closure leading to a diverticulum extending from the fetal midgut to the yolk sac [6]. While both abnormalities are considered rare in patients with Patau’s syndrome, and it is rare for these two congenital defects to appear simultaneously, this case report describes a newborn with Patau’s syndrome who presented with both an omphalocele and an omphalomesenteric duct remnant. 

## Case presentation

**Figure 1:**
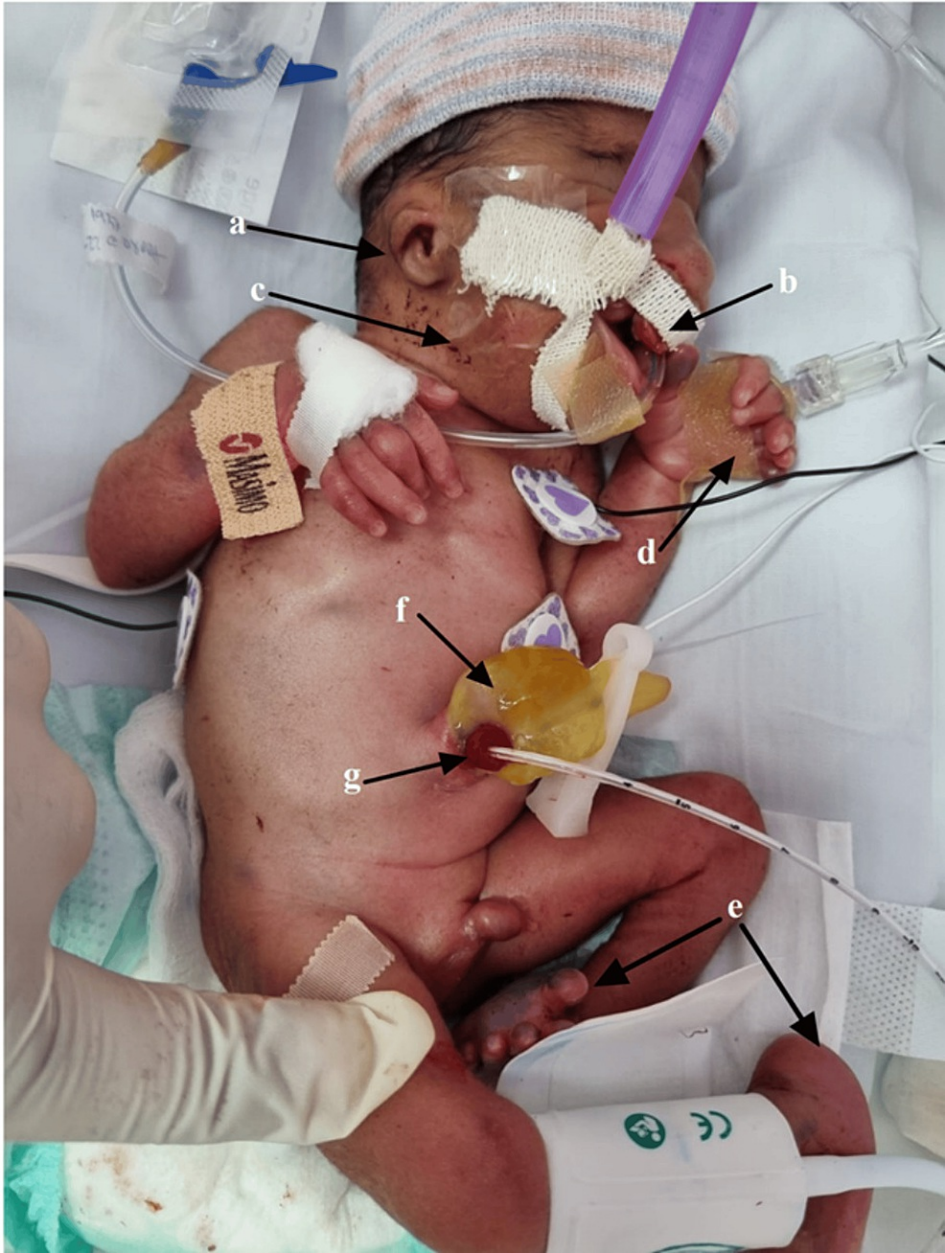
Abnormal features are observed, including low-set ears (a), cleft lip (b), micrognathia (c), polydactyly in the left hand (d), and lower limb deformities (rocker bottom and clubbed foot) (e). The intestinal content is visible through the omphalocele sac (f), and the patent omphalocele duct (g) is shown to be cannulated with a feeding tube to establish the diagnosis.

A 31-week-old Saudi male preterm was delivered through an emergency cesarean section to a 23-year-old mother (Gravida 2, para 2) on March 25, 2023. The mother had previously received two doses of dexamethasone. However, because the baby was preterm and due to his breech position, an emergency cesarean section was performed. The newborn had an APGAR score of 6 in the first minute of life and a score of 7 in the first 5 minutes. Physical examination revealed multiple congenital abnormalities, including coloboma, microphthalmia, broad nasal bridge, cleft lip, cleft palate, low-set ears, polydactyly, rocker-bottom feet, and left-sided clubbed feet (Figure 1). Abdominal examination revealed a small omphalocele containing a bowel covered with a sac arising from the umbilicus and a continuous vitelointestinal remnant on the right side of the omphalocele (omphalomesenteric duct remnant) (Figure 1).

**Figure 2:**
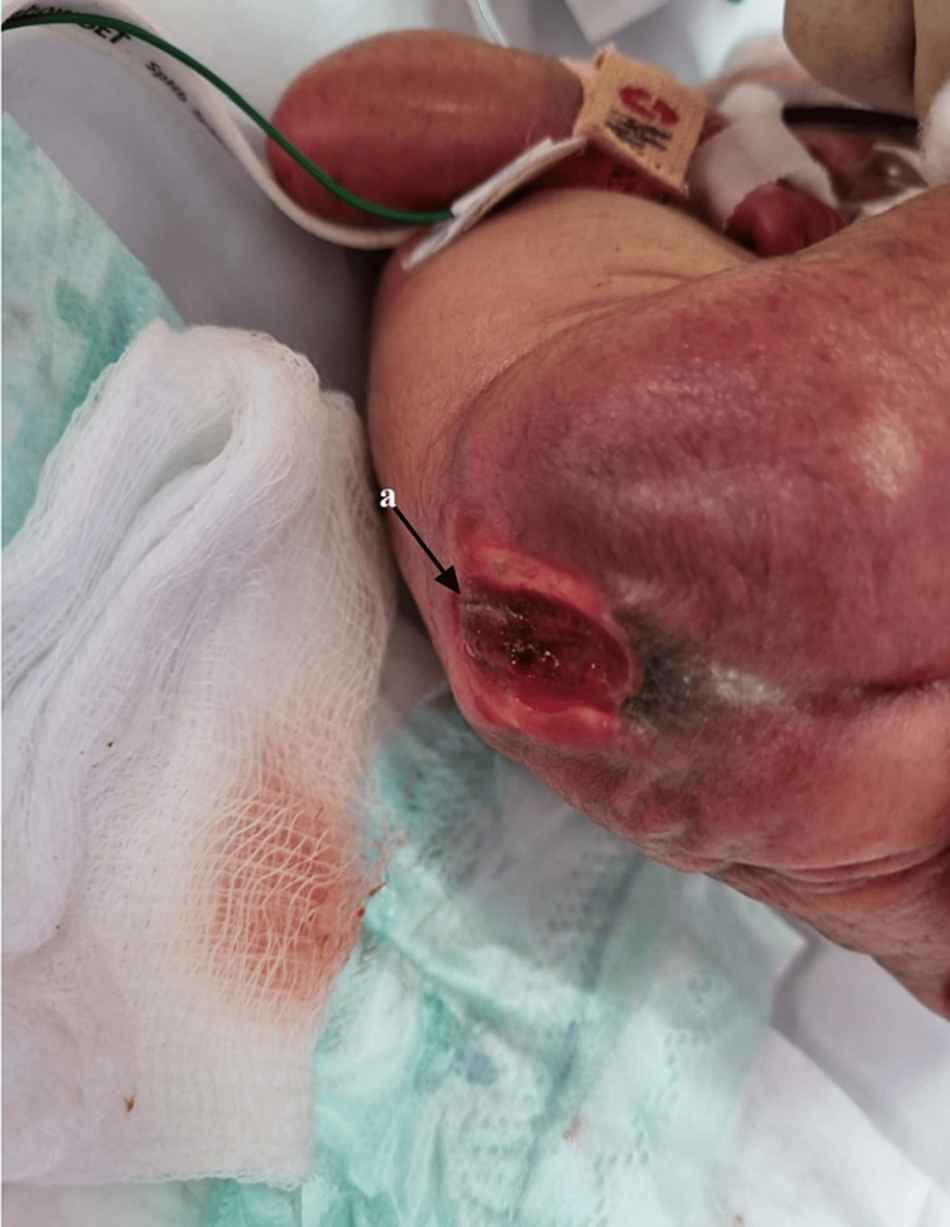
Lower back ruptured myelomeningocele (a).

A ruptured myelomeningocele was also found when examining the patient's back (Figure 2).

**Figure 3:**
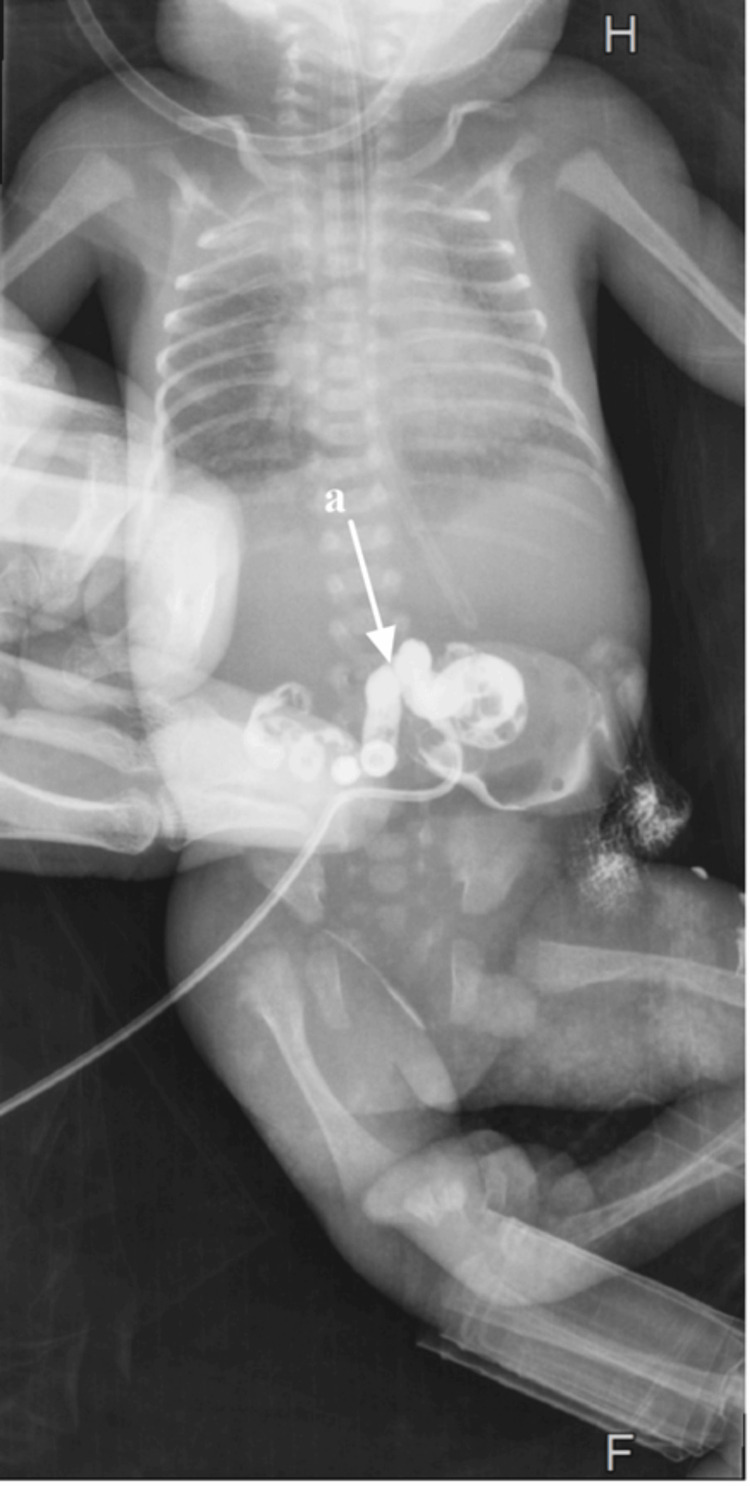
Radiographs with contrast show the continuity of the omphalomesenteric defect with the gastrointestinal tract (a).

The patient was admitted to the neonatal intensive care unit and underwent whole-exome sequencing on March 27, 2023. Respiratory support and intubation with mechanical ventilation were initiated due to respiratory distress syndrome. To rule out sepsis, cefotaxime and gentamycin were administered, and blood cultures were taken and showed no bacterial growth. Laboratory results were unremarkable. Abdominal and pelvic radiographs were obtained after injecting a diluted contrast agent through the cannulated omphalomesenteric defect, confirming the diagnosis (Figure 3).

Head ultrasonography revealed cerebellar hypoplasia. Abdominal ultrasonographic findings were unremarkable. Echocardiography revealed a large ventricular septal defect, a large patent ductus arteriosus, a hypoplastic distal arch, and a markedly dilated right atrium and left ventricle. Chromosomal analysis of the peripheral blood was performed due to the patient’s dysmorphic features, and it was determined that the patient had Patau’s syndrome (Translocation Type) 46 XY +13 defect (13;14) (q10;q10). 

During the hospital stay, the patient experienced thrombocytopenia and received a platelet transfusion. Additionally, he developed renal impairment, as indicated by elevated urea and creatinine levels. Multiple medical teams, including pediatric surgery, neurosurgery, medical genetics, and maxillofacial surgery, were actively involved in the patient’s care. Considering the presence of multiple congenital anomalies, poor prognosis, prematurity, and severe growth retardation, a "Do-Not-Resuscitate" (DNR) decision was made due to his severe comorbidities, and palliative measures were implemented. However, the patient passed away seven days after birth.

## Discussion

The diagnostic criteria for Patau’s syndrome involve identifying certain clinical defect triads such as microphthalmia, polydactyly, and cleft palate. If these are present, a cytogenic analysis is usually performed for confirmation, along with genetic counseling [7].

Patau’s syndrome is associated with an increased rate of spontaneous abortions, in addition to having a median age of survival of approximately two years. However, there are a few exceptions, with some patients living for more than one year or more than a decade [8-12]. Therefore, rigorous treatment should be considered and prompted, such as oxygenation for cardiac abnormalities, nasogastric tube insertion to prevent aspiration, prophylactic medications such as antibiotics to prevent infections, and diagnostic imaging/tests to identify any structural or chromosomal abnormalities to determine the patient’s suitability for surgery [13].

Consistent with other studies, our patient had no cytogenetic abnormalities. However, facial characteristics of Patau’s syndrome, including cleft lip, cleft palate, and low-set ears, similar to those reported in the literature, were present, as were cardiovascular defects such as ventricular septal defects and patent ductus arteriosus [14]. Furthermore, other studies have reported that approximately half of the patients with Patau’s syndrome present with colobomas, cataracts, and microphthalmia upon ocular examination [15]; however, the patient in our case presented with coloboma and microphthalmia without cataracts. The renal ultrasound findings were unremarkable, although approximately 30% of patients with Patau’s syndrome present with renal pathologies [16,17]. Additionally, the patient presented with multiple limb defects, consistent with the findings of other studies [17].

During fetal development, the abdominal wall is relatively small, leading to both the midgut and hindgut herniating around the 5th and 7th weeks of gestation. This herniation typically resolves by the 11th week. Generally, an omphalocele can occur if there is abnormal lateral folding of the gut structures and if the herniation does not return to the umbilicus [18]. Moreover, if the regression of the omphalomesenteric duct fails, it forms Meckle’s diverticulum, the omphalomesenteric continuous vitelointestinal duct, fibrous bands, cysts, and umbilical polyps [18].

There have been case reports, such as those by Lodhia et al. and Abdalkarem et al., where patients presented with a perforated Meckle’s diverticulum in an omphalocele without Patau’s syndrome. However, none of them underwent chromosomal analyses [19,20]. In these abovementioned cases, patients were surgically treated via resection of the perforated Meckle’s diverticulum and end-to-end anastomosis, and the omphalocele was repaired [19,20]. This suggests that there is a high likelihood of perforation when Meckle’s diverticulum and omphalocele are present simultaneously, despite being rare, making surgery the standard of care.

However, in the present case, the patient did not undergo surgery and was placed on a DNR because of multiple comorbidities. As a result, the surgical team focused on stabilizing the patient as much as possible and provided palliative care till the patient’s death. 

Contrary to the above, a case reported by Kotinatot et al. involved a patient with Patau’s syndrome who experienced intermittent volvulus with obstruction due to Meckel’s diverticulum and a peritoneal band extending from the base of the diverticulum to the umbilicus [21]. This patient shared similar characteristics with the current case, including Patau’s syndrome, structural cardiac abnormalities, and cerebellar hypoplasia. The patient underwent surgery, and his condition improved considerably [21].

## Conclusions

Surgical treatment of both the omphalocele and omphalomesenteric duct remnants is considered definitive management to prevent further complications and worsening of the disease if the patient is in a stable state. However, for unstable patients with multiple comorbidities who are deemed unsuitable for surgical management, palliative care is often recommended. Genetic counseling is an essential part of management, regardless of whether patients undergo surgery, as it helps provide a balanced approach to address paternal autonomy, provide a better understanding of the disease and its importance, provide possible alternative methods of managing the disease, and make crucial decisions regarding family care and planning in the future.
